# The Moderating Role of Social Support in the Relationship Between Diabetes Distress and Psychological Well‐Being Among Older Adults With Type 2 Diabetes: A Cross‐Sectional Study Protocol

**DOI:** 10.1002/hsr2.73427

**Published:** 2026-10-27

**Authors:** Elham Imani, Matin Roosta‐ye Abkenar

**Affiliations:** ^1^ Mother and Child Welfare Research Center Hormozgan University of Medical Sciences Bandar Abbas Iran; ^2^ Department of Nursing, Faculty of Nursing and Midwifery Hormozgan University of Medical Sciences Bandar Abbas Iran

**Keywords:** aged, diabetes distress, nursing, older adults, psychological well‐being, social support, type 2 diabetes mellitus

## Abstract

**Background and Aims:**

Type 2 diabetes imposes a considerable psychological burden on older adults, including elevated diabetes distress and diminished psychological well‐being. Social support may buffer the harmful mental‐health effects of chronic disease‐related stress, yet whether it moderates the link between diabetes distress and psychological well‐being remains rarely examined among older Iranian adults with type 2 diabetes. This protocol aims to determine the moderating role of social support in that relationship in this population.

**Methods:**

This descriptive‐analytic cross‐sectional protocol will recruit 300 adults aged 60 years or above with type 2 diabetes from the Bandar Abbas Diabetes Clinic through simple random sampling from the clinic electronic registry. Trained interviewers will administer a demographic and clinical form, the age‐adjusted Charlson Comorbidity Index, the PAID‐5 (diabetes distress), the MSSS‐5 (social support), and the WHO‐5 Well‐Being Index in person or by telephone using identical structured interviews. Hierarchical multiple regression will test the diabetes distress by social support interaction on WHO‐5 scores, in an unadjusted model and a model adjusted for a pre‐specified demographic and clinical covariate set, with heteroscedasticity‐robust standard errors, bootstrapped confidence intervals, and simple‐slopes and Johnson‐Neyman probing. Missing data will be handled with multiple imputation. Analyses will use IBM SPSS Statistics version 28 with two‐sided tests at *α* = 0.05.

**Results:**

As a protocol, no empirical results are reported. The completed study will yield estimates of diabetes distress, social support, and psychological well‐being levels, their bivariate associations, and the moderation effect with appropriate uncertainty quantification.

**Conclusion:**

The findings are expected to provide a scientific basis for nursing and psychosocial interventions that strengthen social support networks to protect the psychological well‐being of older adults with type 2 diabetes. Ethical approval has been obtained (IR.HUMS.REC.1405.013); written or audio‐recorded verbal informed consent will be obtained from all participants.

## 1 Introduction

Type 2 diabetes mellitus remains one of the most burdensome chronic non‐communicable diseases of the twenty‐first century for health systems worldwide. According to the eleventh edition of the International Diabetes Federation (IDF) Diabetes Atlas (2024), about 589 million adults currently live with diabetes globally, a figure projected to reach 853 million by 2050 [1]. In Iran, diabetes prevalence among adults aged 20–79 years was estimated at roughly 9% in 2024, corresponding to approximately 5.4 million individuals [2]. Adults aged 60 years or above form an especially vulnerable subgroup; recent Iranian epidemiologic studies indicate that more than one‐third of this age group lives with type 2 diabetes [3, 4]. Despite broad access to health services, only 41% of Iranian patients with diabetes achieve adequate glycemic control [5], pointing to a gap in comprehensive disease management that extends beyond medical care into its psychological and social dimensions.

Diabetes distress, the emotional burden stemming from everyday disease‐management demands, worries about complications, perceived treatment constraints, and socioeconomic pressures, has become a central psychological construct in the diabetes literature [6]. A substantial body of research links higher diabetes distress with poorer glycemic control, lower treatment adherence, and greater depression risk [7, 8], and among older Iranian adults it has been identified as a principal predictor of reduced quality of life [9]. Psychological well‐being, by contrast, comprising happiness, a sense of meaning, personal growth, and positive relations with others [10], is a positive marker of mental health. Greater well‐being is associated with steadier self‐care, better glycemic control, and fewer diabetes‐related complications [11], and longitudinal evidence from the United Kingdom suggests that individuals with higher pre‐diagnosis well‐being are about 40% less likely to develop depression after a diabetes diagnosis [12].

Social support, encompassing emotional, informational, and instrumental dimensions, has been proposed as an important influence on the association between diabetes distress and psychological well‐being [13]. In the stress‐buffering model of Cohen and Wills, social support attenuates the mental‐health consequences of chronic stressors [14], and empirical work has linked stronger support with lower diabetes distress and better psychological well‐being [15]. In the Iranian context, Parviniannasab and colleagues (2024) reported moderating and mediating roles of social support in the association between diabetes distress and resilience among patients with type 2 diabetes [16], and recent evidence from the United States likewise indicates that social support moderates the association between diabetes distress and quality of life [17].

Despite these advances, most existing studies—including those conducted in Iran—have focused on general adult populations with diabetes rather than on older adults, who face the compounded challenges of aging and comorbidity. In addition, the dependent variables examined in prior research have predominantly centered on the absence of pathology (e.g., depressive symptoms) or on resilience, rather than on the positive construct of psychological well‐being that is the focus of the present study. Consequently, a notable research gap exists regarding the moderating role of social support in the relationship between diabetes distress and psychological well‐being specifically among older Iranian adults with type 2 diabetes.

Given the rapidly aging population of Iran—projected to reach a 16% share of older adults by 2036/1415 [18]—together with the high prevalence of diabetes in this age group and the potentially protective role of psychosocial factors, the present study aims to determine the moderating role of social support in the relationship between diabetes distress and psychological well‐being among older Iranian adults with type 2 diabetes. The findings are expected to provide a scientific basis for designing evidence‐based nursing and psychosocial interventions to promote the psychological well‐being of older adults with type 2 diabetes. Because recruitment is confined to a single referral clinic, the generalizability of the findings beyond this setting will be explicitly examined and reported as a limitation.

### 1.1 Aims and Research Questions

**General aim:** To determine the moderating role of social support in the relationship between diabetes distress and psychological well‐being among older Iranian adults with type 2 diabetes.

Specific Objectives:

1. To determine the levels of diabetes distress, social support, and psychological well‐being among older adults with type 2 diabetes.
2. To determine the relationship between diabetes distress and psychological well‐being among older adults with type 2 diabetes.
3. To determine the relationship between social support and diabetes distress among older adults with type 2 diabetes.
4. To determine the relationship between social support and psychological well‐being among older adults with type 2 diabetes.
5. To determine the moderating role of social support in the relationship between diabetes distress and psychological well‐being among older adults with type 2 diabetes.

Research Questions:

1. What are the levels of diabetes distress, social support, and psychological well‐being among older adults with type 2 diabetes?
2. Is there a significant relationship between diabetes distress and psychological well‐being?
3. Is there a significant relationship between social support and diabetes distress?
4. Is there a significant relationship between social support and psychological well‐being?
5. Does social support moderate the relationship between diabetes distress and psychological well‐being; that is, is there a significant interaction effect among these three variables?

The primary endpoint is the diabetes distress × social support interaction term predicting WHO‐5 scores; objectives 1–4 (descriptive levels and bivariate associations) are secondary. All primary and sensitivity analyses are pre‐specified below to avoid multiplicity inflation.

## 2 Methods and Materials

### 2.1 Study Design

This study will be a descriptive‐analytic, cross‐sectional investigation employing a correlational design to examine the moderating role of social support in the relationship between diabetes distress and psychological well‐being among older adults with type 2 diabetes. This protocol was prepared in accordance with the STROBE guideline for cross‐sectional studies. All primary, adjusted, and sensitivity analyses specified under Statistical Analysis are pre‐specified; any additional analysis will be explicitly labeled exploratory.

### 2.2 Study Setting

The study will be conducted at the Bandar Abbas Diabetes Clinic, Bandar Abbas, Hormozgan Province, Iran, a referral center providing outpatient care to patients with diabetes mellitus. Following approval of the final protocol and issuance of an ethics approval code by the Ethics Committee of Hormozgan University of Medical Sciences, formal permission will be obtained from the Vice‐Chancellery for Research and Technology of the university and communicated to the clinic administration prior to the commencement of data collection.

### 2.3 Participants

The target population will comprise all older adults aged 60 years or above with a confirmed diagnosis of type 2 diabetes mellitus of at least 6 months' duration who attend the Bandar Abbas Diabetes Clinic during the study period (2026–2027).

### 2.4 Eligibility Criteria

**Inclusion criteria:** (1) age ≥ 60 years [19]; (2) confirmed diagnosis of type 2 diabetes mellitus for at least 6 months; (3) an active medical record with at least one clinic visit within the preceding 3 months; (4) ability to communicate in Persian and respond to interview questions; and (5) provision of informed consent to participate [20].

**Exclusion criteria:** (1) a documented history of diagnosed cognitive impairment or acute psychiatric illness (e.g., schizophrenia or major depressive episode) in the medical record; (2) hospitalization within the preceding 3 months; and (3) completion of fewer than 80% of questionnaire items [21].

### 2.5 Recruitment

A complete list of patients aged 60 years or above with type 2 diabetes will be extracted from the clinic electronic medical record system to form the sampling frame. The sampling frame comprises approximately 1672 patient records, including about 550 regularly attending patients. From this frame, 300 participants will be selected using a random‐number‐generation function, and additional randomly ordered replacements will be drawn to allow for non‐response so that 300 completed interviews are achieved. Selected individuals will be contacted by telephone (up to three attempts at different times of day) and invited to attend the clinic, or, where feasible, approached during a scheduled routine visit. To document response and assess non‐response bias, the numbers of contacted, enrolled, and completed interviews will be recorded, basic demographics of responders will be compared with the sampling frame, and post‐stratification weights will be applied if feasible. Prior to enrollment, the study aims, procedures, confidentiality safeguards, and voluntary nature of participation will be explained in full, and written informed consent (or audio‐recorded verbal consent for telephone enrollment, witnessed and documented) will be obtained after a full explanation of the study purpose, procedures, and the right to withdraw at any stage without consequence for clinical care. For participants unable to attend the clinic because of physical limitations, an identical structured interview with the same item wording and order will be administered by telephone. Data‐collection mode (in‐person vs*.* telephone) and interviewer identity will be recorded for every interview as methodological covariates.

Simple random sampling, rather than a full census of eligible patients, was selected because a census of all patients ≥ 60 years with type 2 diabetes attending the clinic and affiliated comprehensive health centers would require substantially greater time and resources than are available within the study's 7‐month timeframe. Simple random sampling reduces operational burden while ensuring that every eligible patient has an equal probability of selection, thereby preserving the representativeness of the target population. A sample of 300 is expected to provide adequate statistical power (≥ 0.80) to detect small‐to‐moderate effect sizes in the hierarchical regression analysis, while remaining cost‐effective. In addition, the extraction of demographic and clinical data from medical records, combined with interviewer‐administered data collection, is intended to enhance data accuracy and reduce self‐report bias. To protect the target of 300 completions against an anticipated non‐response of up to roughly 30%, the randomized contact list will include ordered replacements drawn by the same random procedure.

### 2.6 Sample Size

The required sample size was calculated using the standard formula for correlational studies, assuming a 95% confidence level, 80% statistical power, and a minimum expected correlation coefficient of *r* = 0.25, based on comparable prior studies:

*n* = [(Zα/2 + Zβ)/(0.5 × ln((1 + r)/(1 – r)))]^2^ + 3

Substituting Zα/2 = 1.96, Zβ = 0.84, and *r* = 0.25 yields a base sample size of approximately 245 participants. Accounting for an anticipated attrition/non‐response rate of 15%, the adjusted sample size is 285 participants; given the additional statistical power required for the hierarchical moderation analysis, the final target sample size was set at 300 participants [22].

Dedicated power for the interaction term. Beyond the bivariate calculation above, a priori power for the primary diabetes distress × social support interaction was evaluated for a multiple‐regression *R* ^2^‐increase test (one tested predictor; G*Power 3.1 procedure [23]). At *N* = 300 with two‐sided *α* = 0.05 and the full pre‐specified covariate set, power reaches ≥ 0.80 for small‐to‐moderate incremental interaction effects (Cohen *f* ^2^ ≈ 0.03 and above [22]); smaller effects will be interpreted through effect sizes and confidence intervals rather than *p* values alone.

### 2.7 Variables

#### 2.7.1 Measurement Instruments

##### 2.7.1.1 Demographic and Clinical Data Form

A researcher‐developed form will capture demographic characteristics (age, sex, marital status, education level, employment status, monthly household income, insurance coverage, living arrangement, number of children, family history of diabetes, smoking status, and physical activity level) and clinical characteristics extracted from medical records (diabetes duration, body mass index, most recent glycated hemoglobin [HbA1c] value, and documented diabetes‐related complications).

##### 2.7.1.2 Age‐Adjusted Charlson Comorbidity Index (ACCI)

Overall comorbidity burden will be assessed using the Charlson Comorbidity Index adjusted for age, a 17‐item weighted checklist of chronic conditions (e.g., cerebrovascular disease, congestive heart failure, renal disease) combined with an age‐based score. Total scores range from 0 to 24, with higher scores indicating greater comorbidity burden and lower expected survival. The Persian version demonstrated acceptable reliability (intraclass correlation coefficient = 0.77) and construct validity against self‐rated health and activities of daily living in a sample of 600 Iranian community‐dwelling older adults [24].

##### 2.7.1.3 Problem Areas in Diabetes Scale, Short Form (PAID‐5)

Diabetes distress will be measured using the 5‐item short form of the Problem Areas in Diabetes scale, which assesses emotional burden related to diabetes management, including fear of complications, diabetes‐related depressive feelings, and treatment‐related fatigue. Each item is rated on a 5‐point Likert scale (0 = not a problem to 4 = a very serious problem), yielding raw scores from 0 to 20; scores of 8 or above indicate clinically significant diabetes distress. The Persian version was validated among 110 patients with diabetes attending a diabetes clinic in Dorud, Iran, demonstrating strong correlation with the full 20‐item version (*r* = 0.887) and acceptable internal consistency (Cronbach's *α* = 0.75), with a screening cut‐off of 8 [18]. Cronbach's α and McDonald's ω for the PAID‐5 will be estimated in the study sample and reported; the implications of short‐form reliability for attenuation of the interaction effect will be discussed.

##### 2.7.1.4 Medical Outcomes Study Social Support Survey, Short Form (MSSS‐5)

Perceived social support will be assessed using the 5‐item short form of the Medical Outcomes Study (MOS) Social Support Survey, which captures emotional support (e.g., having someone to confide in) and instrumental support (e.g., help during illness). Items are rated on a 5‐point Likert scale (1 = none of the time to 5 = all of the time), with higher mean scores reflecting greater perceived social support. Among 420 Iranian community‐dwelling older adults, the instrument demonstrated favorable content validity (CVI = 0.94), internal consistency (Cronbach's *α* = 0.78), and temporal stability (ICC = 0.98), with confirmatory factor analysis supporting its two‐factor (emotional and instrumental support) structure [25]. The pre‐specified moderator is the MSSS‐5 overall mean score; the established emotional (3 items) versus instrumental (2 items) structure is noted descriptively, and no separate subscale moderation tests are pre‐specified as primary. Cronbach's α and McDonald's ω will be estimated in‐sample and reported.

##### 2.7.1.5 WHO‐5 Well‐Being Index

Subjective psychological well‐being over the preceding 2 weeks will be assessed using the WHO‐5 Well‐Being Index, a brief, non‐intrusive instrument comprising five positively worded items (e.g., “I have felt calm and relaxed”) rated on a 6‐point Likert scale (0 = at no time to 5 = all of the time). Total scores range from 0 to 25, with scores below 13 indicating low well‐being and warranting further evaluation for depressive symptoms. The Persian version was validated in a sample of 498 Iranian university students, demonstrating a unidimensional factor structure, internal consistency (Cronbach's *α* = 0.89), test–retest reliability (0.82), convergent validity with the Oxford Happiness Questionnaire (*r* = 0.57), and divergent validity with the Beck Depression Inventory (*r* = –0.61) [26]. Because this Persian validation was conducted among university students, construct validity in older adults with diabetes cannot be assumed; Cronbach's α and McDonald's ω will be estimated in‐sample, and supplemental validity evidence (confirmatory factor structure and known‐groups comparisons) will be examined and reported as feasible for this cohort, with any limitations discussed.

### 2.8 Data Collection Procedure

Following ethics approval and formal introduction to the Bandar Abbas Diabetes Clinic, data collection will proceed as follows. A complete list of eligible patients will be extracted from the electronic medical record system, and 300 participants will be selected using random‐number generation in SPSS, with randomly ordered replacements to allow for non‐response. Selected individuals will be contacted by telephone to schedule a clinic visit or approached during a routine visit. After a full explanation of the study purpose, procedures, confidentiality safeguards, voluntary participation, and the right to withdraw without consequence for care, written informed consent (or audio‐recorded verbal consent for telephone enrollment) will be obtained from every participant before any data are collected. Participants will complete the questionnaires in a quiet, low‐stimulation training room in a single session of about 15–20 min (divided into two shorter sittings if needed). To reduce cognitive burden and missing data, a trained undergraduate nursing student, supervised by the principal investigator, will administer all items verbally in a consistent neutral tone and record responses verbatim without interpretation. Procedural safeguards against common‐method variance include a calm private setting, neutral interviewing, brief validated instruments, identical in‐person and telephone scripts, and supplementation of demographic and clinical variables from medical records. Mode and interviewer identity are recorded for covariate adjustment and sensitivity analyses.

If, during data collection, a participant's diabetes distress score exceeds 8 on the PAID‐5 or their WHO‐5 well‐being score falls below 13, the participant will be referred, in accordance with the study's ethical protocol, to the psychological counseling unit based at the clinic.

### 2.9 Data Management

All collected data will be coded and entered into IBM SPSS Statistics version 28. Data quality control will include screening for missing values, correction of entry errors, and outlier detection using box plots and standardized z‐scores (|z | > 3.29). Missing‐data patterns and mechanisms will be reported (proportions per variable, Little's MCAR test, and comparison of complete *vs.* incomplete cases). The default handling will be multiple imputation with chained equations (fully conditional specification, *m* = 20 imputed data sets) under a missing‐at‐random assumption, with estimates pooled using Rubin rules; a complete‐case analysis will be reported as a sensitivity check. Mean imputation will not be used.

#### 2.9.1 Anonymization

Each participant will be assigned a unique, sequential study identification code at the time of enrollment. All analysis datasets will contain only this code; personal identifiers (name, national ID, and contact details) will be stored in a separate linkage file accessible only to the principal investigator, for the sole purpose of facilitating referral to the psychological counseling unit when clinically indicated.

#### 2.9.2 Storage and Access Control

Electronic data will be stored on a password‐protected, institution‐managed computer at the School of Nursing and Midwifery, Hormozgan University of Medical Sciences, with access restricted to named members of the research team (the principal investigator, co‐investigators, and the biostatistics consultant). Paper‐based consent forms and any physical records will be kept in a locked cabinet accessible only to the principal investigator. The participant identifier linkage file will be encrypted and stored separately from the analysis dataset.

#### 2.9.3 Data Retention

Study data will be retained for 5 years following completion of the study, in accordance with the data retention policy of Hormozgan University of Medical Sciences, after which records will be securely destroyed.

#### 2.9.4 Confidentiality

Data will be reported exclusively in aggregate, de‐identified form in all publications and presentations. No individually identifiable information will be disclosed to any party outside the research team.

### 2.10 Statistical Analysis

Descriptive statistics (means and standard deviations for continuous variables; frequencies and percentages for categorical variables) will characterize demographic, clinical, and study variables. Prior to inferential analyses, regression assumptions will be checked: normality of residuals (Kolmogorov–Smirnov test with Q–Q plots), linearity (scatterplots), homoscedasticity, independence of residuals (Durbin–Watson statistic; acceptable range 1.5–2.5), and multicollinearity (tolerance > 0.1; variance inflation factor < 10). Internal consistency (Cronbach's α and McDonald's ω) will be estimated in‐sample for the PAID‐5, MSSS‐5, and WHO‐5. All tests are two‐sided at *α* = 0.05. Effect sizes with 95% confidence intervals are reported alongside P values, which are never presented alone; *p* values below 0.001 are reported as *p* < 0.001, values 0.001–0.01 to the nearest thousandth, values ≥ 0.01 to the nearest hundredth, and values above 0.99 as *p* > 0.99 [27].

Bivariate associations among diabetes distress (PAID‐5), social support (MSSS‐5), and psychological well‐being (WHO‐5) will be examined with Pearson correlation coefficients. The primary hypothesis will be tested with hierarchical multiple regression. Predictors will be mean‐centered before computing the interaction term. In the unadjusted (primary) model, diabetes distress and social support enter first as main effects, then the diabetes distress × social support interaction enters to evaluate incremental variance (Δ*R* ^2^). Heteroscedasticity‐robust (HC3) standard errors will be estimated and the interaction effect bootstrapped (5000 resamples) to guard against non‐normality and heteroscedasticity. A significant interaction will be probed with simple slopes at one standard deviation above and below mean support, Johnson–Neyman regions of significance, and a plot of conditional effects. Standardized (β) and unstandardized (B) coefficients, 95% confidence intervals, *F* statistics, *R* ^2^/Δ*R* ^2^, and Cohen *f* ^2^ for the interaction [22] will be reported for each model.

An adjusted model will then be estimated with a fully pre‐specified covariate set chosen to reduce omitted‐variable bias and prevent post‐hoc modeling flexibility: age, sex, marital status, education, employment status, monthly household income, insurance coverage, living arrangement, number of children, family history of diabetes, smoking status, physical activity level, body mass index, HbA1c, diabetes duration, documented diabetic complications, the age‐adjusted Charlson Comorbidity Index, data‐collection mode, and interviewer identity. These cover the broad demographic and clinical variables of Table 1; mode and interviewer address systematic measurement variance. Because the ACCI incorporates age weighting, collinearity between age and the ACCI will be checked via variance inflation factors; if VIF ≥ 10, the pre‐specified fallback retains the ACCI and drops standalone age, reported transparently. Covariates enter first, followed by the main effects and finally the interaction term; consistency of the interaction across unadjusted and adjusted models assesses robustness. Mode‐stratified (in‐person vs. telephone) and interviewer‐adjusted sensitivity analyses are pre‐specified, as is a Harman single‐factor check for common‐method variance. The primary endpoint is the interaction term; descriptive and bivariate objectives are secondary, and all pre‐specified analyses will be reported transparently without formal multiplicity correction across sensitivity checks, emphasizing confidence intervals over isolated *p* values. Statistical procedures comprise descriptive statistics, Pearson correlations, and hierarchical multiple regression with mean‐centering, HC3 robust standard errors, bootstrap resampling, simple‐slopes and Johnson–Neyman probing, Little's MCAR test, and multiple imputation by chained equations, performed in IBM SPSS Statistics version 28.

**Table 1:** Summarizes the study variables, their operational roles, and measurement levels.

Variable	Role	Type/Level	Instrument
Diabetes distress	Independent variable	Quantitative (continuous)	PAID‐5
Social support	Moderator variable	Quantitative (continuous)	MSSS‐5
Psychological well‐being	Dependent (outcome) variable	Quantitative (continuous)	WHO‐5
Age, sex, education, marital status, employment, income, insurance coverage, living arrangement, number of children, family history of diabetes, smoking, physical activity	Covariates	Mixed (quantitative/qualitative)	Demographic and clinical questionnaire
BMI, HbA1c, diabetes duration, diabetic complications	Covariates	Quantitative/qualitative	Medical record extraction
Comorbidity burden (age‐adjusted)	Covariate	Quantitative (continuous)	ACCI
Data collection mode (in‐person/telephone)	Methodological covariate	Qualitative (binary)	Study records
Interviewer identity	Methodological covariate	Qualitative	Study records

### 2.11 Ethical Considerations

This study has been approved by the Research Ethics Committee of Hormozgan University of Medical Sciences (approval code: IR.HUMS.REC.1405.013) and has received formal authorization from the university's Vice‐Chancellery for Research and Technology. Written or audio‐recorded verbal informed consent will be obtained from all participants after a full explanation of the study's purpose, procedures, and voluntary nature; participants will be informed of their right to withdraw at any stage without consequence for their clinical care. Participants identified during data collection as having clinically significant diabetes distress (PAID‐5 ≥ 8) or low psychological well‐being (WHO‐5 < 13) will be referred to the psychological counseling unit at the clinic in accordance with the approved ethical protocol. No pharmacological or invasive interventions will be administered as part of this study. As this is a non‐interventional, observational cross‐sectional study, registration with a clinical trial registry (e.g., IRCT) is not applicable; no participants are assigned to interventions or comparison groups. Consent is obtained in a private setting with opportunity for questions; for telephone enrollment, verbal consent is audio‐recorded, witnessed, and documented in the study log.

## 3 Discussion/Expected Contributions

This protocol describes a cross‐sectional study designed to examine whether social support moderates the relationship between diabetes distress and psychological well‐being among older adults with type 2 diabetes in Iran. Based on the stress‐buffering model [14] and prior empirical evidence [15, 16, 28], it is anticipated that higher levels of social support will attenuate the adverse effect of diabetes distress on psychological well‐being. Should this hypothesis be supported, the findings may contribute evidence relevant to the design of nursing and psychosocial interventions aimed at strengthening social support networks for older adults with diabetes, particularly within the context of routine diabetes clinic care.

Unlike much of the existing literature, which has predominantly examined general adult populations with diabetes and outcomes framed around the absence of pathology (e.g., depressive symptoms), this study focuses specifically on older adults and on psychological well‐being as a positive mental health construct. As such, the findings could inform a more strengths‐based approach to psychosocial care for this population and may help identify subgroups of older patients—those reporting high diabetes distress alongside low perceived social support—who could benefit most from targeted supportive interventions.

The results may also have relevance for health policy, potentially informing the development of community‐based social support programs for older adults with chronic illness within Iran's evidence‐based health system. However, given the cross‐sectional design, causal inferences regarding the direction of the relationships examined cannot be drawn, and the findings should be interpreted as describing associations at a single point in time.

Interpretation will prioritize effect sizes and confidence intervals over isolated P values [27]. The moderation inference rests on the pre‐specified interaction term, probed and visualized as above; adjusted and sensitivity models test its robustness rather than generating new primary claims.

### 3.1 Study Limitations

#### 3.1.1 Limited Participant Availability

Some older adults may be unwilling or physically unable to attend the clinic. This will be addressed by offering telephone‐based interviews for participants unable to attend in person, providing a full explanation of the study's aims and benefits, and collaborating with clinic staff to facilitate participant identification and engagement.

#### 3.1.2 Time Constraints

Older participants may experience fatigue during data collection. This will be mitigated by using brief, standardized instruments, allowing flexible scheduling, and, where necessary, dividing data collection into two shorter sessions.

#### 3.1.3 Social Desirability Bias

Participants may provide socially desirable rather than fully candid responses. This risk will be reduced by emphasizing confidentiality, training interviewers to create a comfortable and non‐judgmental interview environment, and using validated self‐report instruments.

#### 3.1.4 Sample Attrition

Some participants may withdraw during the study. A 15% attrition allowance has been incorporated into the sample size calculation, and regular follow‐up together with appropriate incentives (e.g., individualized feedback on results) will be used to support retention.

Non‐response and single‐clinic scope. Recruitment from one referral clinic limits external validity to similar settings. Response metrics, responder‐versus‐frame comparisons, and any feasible weighting will be reported so the direction of potential non‐response bias can be judged.

Residual mode and interviewer effects. Although mode and interviewer are modeled and stratified, residual systematic variance cannot be fully excluded and will be discussed.

Common‐method variance. Key constructs are self‐reported in one session; procedural safeguards and the Harman check above mitigate but do not eliminate this concern, which will be acknowledged when interpreting associations.

Short‐form reliability and WHO‐5 validity. Brief instruments constrain reliability and hence interaction detectability; in‐sample α/ω and the WHO‐5 validity evidence in this older cohort will bound this limitation.

## Author Contributions

**Elham Imani:** conceptualization, writing – original draft, writing – review and editing, data curation, methodology. **Matin Roosta‐ye Abkenar:** conceptualization, methodology, investigation, writing – original draft, writing – review and editing, supervision, software, validation.

## Declaration of Generative AI and AI‐Assisted Technologies

During the preparation of this manuscript, the author(s) used Claude (Anthropic‐Sonnet 5 free model) to assist with translating portions of the manuscript from Persian (Farsi) into English. After using this tool, the author(s) reviewed, edited, and verified all translated content for scientific and linguistic accuracy, and take full responsibility for the content of the publication.

## Funding

The authors have nothing to report.

## Ethics Statement

Ethical approval has been obtained from the Research Ethics Committee of Hormozgan University of Medical Sciences (approval code: IR.HUMS.REC.1405.013). Written or audio‐recorded verbal informed consent will be obtained from all participants.

## Consent

The authors have nothing to report.

## Conflicts of Interest

The authors declare no conflicts of interest.

## Transparency Statement

The lead author (MRA) affirms that this manuscript is an honest, accurate, and transparent account of the study protocol being reported, that no important aspects of the study have been omitted, and that any discrepancies from the study as planned will be explained.

Reference verification: at revision (October 2026) all cited works were re‐checked against PubMed and the Retraction Watch database (via Zotero); no retracted publications were retained, and any issued corrections were evaluated for relevance to the citation.

## Data Availability

No datasets were generated or analysed during preparation of this protocol. Data arising from the planned study will be made available from the corresponding author upon reasonable request and subject to institutional and ethics‐committee approval; where data cannot be shared, the restriction will be stated. Study materials and analysis templates will be shared likewise to the extent permitted.
